# A yoga-integrated mind-body movement intervention: effects on children's problem solving, mathematics anxiety, and physical activity

**DOI:** 10.3389/fpubh.2026.1874706

**Published:** 2026-08-06

**Authors:** Derya Kayıran, Alperen Avci, Esra Nur Türkmen, Rahma Yusuf Haji Mohamud

**Affiliations:** 1 Department of Child Development, Vocational School of Health Services, Kahramanmaraş Sütçü Imam University, Kahramanmaraş, Türkiye; 2 Department of Child Development, Malazgirt Vocational School, Muş Alparslan University, Muş, Türkiye; 3 Department of Physiotherapy and Rehabilitation, Vocational School of Health Services, Kahramanmaraş Sütçü Imam University, Kahramanmaraş, Türkiye; 4 Yardimeli Specialist Hospital, Mogadishu, Somalia

**Keywords:** elementary education, embodied learning, mathematics anxiety, movement-integrated curriculum, physical activity, school-based intervention, yoga-integrated learning

## Abstract

**Introduction:**

This study examined the effects of a 10-week yoga-integrated mind-body movement program embedded within routine mathematics instruction on elementary school students' problem-solving performance, computation skills, mathematics anxiety, and self-reported physical activity.

**Methods:**

A quasi-experimental design was employed with third-grade students assigned to either a yoga-integrated mathematics group or a traditional mathematics instruction group. Outcome measures included verbal problem-solving performance, computation skills, overall mathematics achievement, mathematics anxiety, and self-reported physical activity assessed using the Physical Activity Questionnaire for Older Children (PAQ-C).

**Results:**

Compared with the control group, students who participated in the yoga-integrated program demonstrated significantly greater improvements in problem-solving performance, computation skills, and overall mathematics achievement. The intervention group also showed significantly lower mathematics anxiety at post-test. In addition, students in the intervention group reported higher PAQ-C scores following the intervention, suggesting increased self-reported physical activity.

**Discussion:**

Overall, the findings suggest that integrating structured mind-body movement into routine mathematics lessons may support cognitive, emotional, behavioral, and physical aspects of learning. However, because physical activity was assessed using a self-report instrument and the intervention was implemented in intact classrooms within a quasi-experimental design, the findings should be interpreted with appropriate caution. Future studies employing active comparison groups, objective measures of physical activity (e.g., accelerometry), and longitudinal follow-up designs are needed to clarify the mechanisms underlying these associations and to evaluate the long-term effectiveness of yoga-integrated mathematics instruction.

## Introduction

Interest in holistic education models that support children's cognitive, emotional, and physical development has increased markedly in recent years. This rising attention is largely attributed to research demonstrating the substantial physiological and cognitive benefits of physical activity. Indeed, physical activity has been reported to elevate dopamine levels and enhance mental functioning (1). Movement- and meditation-based activities have been shown to reduce state anxiety and improve mood (2). Exercise-based interventions are also supported by substantial evidence demonstrating their positive effects on intelligence development, cognitive processes, and academic performance (3, 4). Furthermore, robust evidence has demonstrated that physical activity contributes significantly to motor and cognitive functions, social behaviors, and emotional regulation (5, 6). Short bouts of rhythmic exercise have been shown to enhance visual-motor integration and behavioral regulation, while components of physical fitness are reported to be closely associated with neurocognitive processes, attention, executive functioning, and academic performance (7, 8). Large-scale reviews indicate that physical activity supports core academic skills such as reading, mathematics, and foreign language learning, and numerous studies report that classroom-based physical activity breaks contribute markedly to academic motivation, attention span, task completion, and mathematics performance (9–13). Moreover, aerobic exercise programs have been reported to enhance academic engagement and academic resilience (14). Collectively, these findings demonstrate that physical activity enhances not only physical health but also the cognitive and emotional processes that underlie learning.

These findings are also closely aligned with contemporary curriculum and instructional design perspectives that emphasize the integration of academic learning, health promotion, and whole-child development within routine school practices. Rather than treating cognitive achievement, emotional wellbeing, and physical development as separate educational domains, current curriculum frameworks increasingly advocate learning environments that address these dimensions simultaneously. The Whole School, Whole Community, Whole Child model, for example, conceptualizes learning and health as interconnected components of educational attainment and healthy development (15–17). Similarly, curriculum integration theory suggests that meaningful learning experiences can be strengthened when disciplinary boundaries are crossed and educational activities are organized around interconnected developmental and instructional goals (18–20). From this perspective, integrating movement-based and self-regulation-oriented activities into mathematics instruction may represent not only a health-supportive classroom practice but also a curriculum innovation that connects academic, emotional, and physical learning objectives. This approach is consistent with the science of learning and development, which emphasizes that children's learning is shaped by interactions among cognitive, social, emotional, and physiological processes (21).

In line with this literature, it is emphasized that providing children with opportunities for movement within educational settings is of critical importance (22). Movement-based education has been reported to provide new perspectives in problem solving, support mental development, and strengthen attentional capacities. However, it has been noted that structured movement programs directly integrated with academic content remain limited (23). In this context, yoga emerges as a distinctive mind-body movement approach that integrates physical postures (asanas), breathing techniques (pranayama), and meditation. Originating from ancient India, yoga encompasses practices aimed at fostering physical, mental, and spiritual harmony, and its global popularity has continued to grow rapidly (24). The designation of the International Day of Yoga by the United Nations has further reinforced its significance (25). Research indicates that yoga meets health-related physical activity criteria, exerts beneficial effects on depression, anxiety, stress, and fatigue, and serves as a core component of mindfulness-based mind-body approaches (26–29). Research on children's yoga demonstrates significant improvements in areas such as flexibility, muscular strength, balance, attention, self-regulation, emotional wellbeing, and social confidence (30–36). Additionally, improvements in working memory, attentional processes, and stress-management skills have also been reported.

Studies integrating physical activity with academic learning remain limited. Although motor-cognitive integrated mathematics instruction programs have been shown to enhance geometry and number learning performance, research examining the integration of yoga-based physical activity into mathematics instruction is exceedingly scarce (37, 38). Yet yoga holds substantial potential in the context of mathematics learning due to its documented effects on attentional focus, anxiety regulation, self-regulation, and working memory (33). In addition, it has been reported that physical inactivity is markedly high among children and adolescents, with 81% of individuals aged 11–17 worldwide failing to meet the recommended levels of physical activity (39). Given that opportunities for physical activity are largely confined to school settings, the incorporation of movement-based approaches into educational programs becomes a necessary priority (40). When the literature is considered holistically, yoga-based physical activity appears to exert strong effects on children's cognitive, emotional, and physical development; however, studies examining its influence on mathematics learning-particularly in relation to problem-solving performance, mathematics anxiety, and physical activity levels-remain exceedingly limited. This gap underscores the need for rigorous scientific investigation into the integration of yoga-based practices, which simultaneously target anxiety regulation, attentional focus, and executive functions, within mathematics instruction. Accordingly, the present study seeks to address this gap by comprehensively examining the effects of yoga-supported mathematics education on children's problem-solving skills, mathematics anxiety, and physical activity levels.

## Materials and methods

This study is a quantitative investigation designed to examine the effects of a yoga-integrated mind-body movement program on children's problem-solving skills, mathematics anxiety, and physical activity levels. A pre-test–post-test quasi-experimental design with a control group was employed to enable a comparative evaluation of the intervention's effectiveness. In quasi-experimental designs, experimental and control groups are established, and pre-intervention (pre-test) and post-intervention (post-test) measurements of the study variables are compared. This design allows for causal inferences similar to those in true experimental studies, yet is preferred in situations where full random assignment to groups is not feasible (41). In this study, full random assignment was not possible due to the natural structure of classroom settings. Therefore, the groups were selected from existing classes, with one designated as the experimental group (receiving the yoga-integrated mind–body program) and the other as the control group (receiving traditional mathematics instruction).

### Study group

The study participants consisted of third-grade students enrolled in a public primary school in Türkiye during the 2023–2024 academic year. Following approval from the school administration, the study was conducted in two intact third-grade classrooms. To preserve the natural classroom structure, individual random assignment was not feasible; therefore, one existing classroom was assigned to the experimental group, which received the yoga-integrated mind–body movement program, whereas the other classroom served as the control group and continued to receive the standard mathematics curriculum.

The study sample comprised 48 students, including 25 students in the experimental group and 23 students in the control group. Participants were between 8 and 9 years of age, and all were regularly attending third-grade mathematics classes. The demographic characteristics of the participants are presented in Table 1.

**Table 1:** Baseline demographic characteristics of the participants.

Characteristic	Experimental (*n* = 25)	Control (*n* = 23)	Total (*N* = 48)
Age (years), Mean ± SD	8.52 ± 0.51	8.48 ± 0.51	8.50 ± 0.51
Female, *n* (%)	13 (52.0)	11 (47.8)	24 (50.0)
Male, *n* (%)	12 (48.0)	12 (52.2)	24 (50.0)
Socioeconomic status, *n* (%)			
Low	7 (28.0)	6 (26.1)	13 (27.1)
Middle	14 (56.0)	13 (56.5)	27 (56.3)
High	4 (16.0)	4 (17.4)	8 (16.6)
Previous year's mathematics grade, Mean ± SD	88.3 ± 5.8	87.5 ± 6.1	87.9 ± 5.9
Regular school attendance (>90%), *n* (%)	25 (100)	23 (100)	48 (100)

Following the examination of baseline demographic characteristics and pre-intervention outcome measures, no meaningful differences were observed between the experimental and control groups before the intervention, indicating that the groups were broadly comparable at baseline. Nevertheless, because the study employed two intact classrooms rather than individual randomization, subsequent analyses controlled for baseline performance using analysis of covariance (ANCOVA) to reduce the potential influence of initial differences.

An a priori power analysis was conducted using G^*^Power 3.1 (42) to determine the minimum sample size required for the study. Assuming a fixed-effects ANCOVA with one covariate, two groups, a large effect size (*f* = 0.40), an alpha level of 0.05, and statistical power of 0.80, the required minimum sample size was estimated to be 46 participants. The final sample consisted of 48 students (25 in the experimental group and 23 in the control group), indicating that the study was adequately powered to detect large intervention effects.

### Data collection instruments

Verbal Problem-Solving Test: In this study, the Verbal Problem-Solving Test developed by Kir (43) was used to assess students' addition-subtraction skills and problem-solving abilities. The test consists of 37 items and comprises two subdimensions: a 22-item Verbal Problem section and a 15-item Computation section. Scores range from 0 to 22 in the Verbal Problem section and from 0 to 15 in the Computation section. In the reliability study reported by Kir (43), internal consistency coefficients were found to be KR-20 = 0.86 for the overall test, 0.89 for the problem-solving section, and 0.82 for the computation section. These results indicate that the instrument is a reliable measurement tool suitable for use in the present study (43).

Mathematics Anxiety Scale: To assess students' levels of mathematics anxiety, the Mathematics Anxiety Scale for 3rd and 4th Grade Students (MAS) developed by Mutlu and Söylemez (44) was used. The scale consists of 13 items formatted on a 3-point Likert scale (agree–undecided–disagree). Positive items are scored as 3-2-1, whereas negative items are reverse-scored as 1-2-3. Total scores range from 13 to 39, with higher scores indicating greater anxiety. The reliability of the scale was evaluated using Cronbach's alpha, and the internal consistency coefficient for the entire scale was reported as α =0.747 (44).

Physical Activity Level: To assess children's physical activity levels, the Physical Activity Questionnaire for Older Children (PAQ-C), adapted into Turkish by Erdim et al. (45), was utilized. The PAQ-C is a self-report instrument designed to evaluate children's moderate-to-vigorous physical activity levels. In the Turkish adaptation study, the content validity index (CVI) was reported as 0.95. Exploratory factor analysis revealed a two-factor structure comprising “in-school activities” and “out-of-school activities,” and this structure was confirmed through confirmatory factor analysis. Factor loadings ranged from 0.41 to 0.80. The scale demonstrated high reliability, with a Cronbach's alpha of 0.77 for the total scale and a test–retest reliability coefficient of ICC = 0.91. These findings indicate that the Turkish version of the PAQ-C is a valid and reliable instrument for assessing children's physical activity levels (45).

### Yoga-integrated mind-body movement program

The yoga-integrated mind-body movement program implemented in this study was delivered over a 10-week periodduring the 2023–2024 spring semester, with three sessions per week, each lasting approximately 20 min. Prior to the intervention, students received age-appropriate explanations regarding the aims of the program, including the potential benefits of yoga for attention, body awareness, breathing regulation, and emotional wellbeing. Participation was entirely voluntary, and all activities were designed to be safe, developmentally appropriate, and suitable for children in the third grade.

The intervention was delivered by the experimental classroom teacher, who had completed formal training in children's yoga before the study. In contrast, the control classroom continued to receive the standard third-grade mathematics curriculum delivered by its regular classroom teacher, without the addition of yoga or other structured movement activities. The intervention and comparison conditions were therefore implemented under routine classroom conditions while preserving the natural organization of the school.

To enhance the consistency of intervention delivery, the yoga-integrated lessons followed a standardized intervention manual consisting of scripted instructions, predefined activities, and fixed session durations. During the development of the program, expert opinions were obtained from specialists in physiotherapy and rehabilitation, child development, and mathematics education to ensure both pedagogical appropriateness and child safety.

Intervention fidelity was monitored throughout the 10-week implementation using a Session Fidelity Checklist, which evaluated adherence to the planned session sequence, activity completion, instructional accuracy, session duration, and safety procedures. Fidelity monitoring indicated that all intervention components were implemented according to the predefined protocol across all sessions.

The program was integrated directly into mathematics instruction and consisted of simple and developmentally appropriate yoga postures, breathing exercises, brief mindfulness and relaxation activities, and playful movement tasks designed to facilitate concentration, sustained attention, emotional regulation, body awareness, and readiness for mathematical problem solving. Activities were intentionally structured to avoid competition or performance pressure, thereby encouraging participation in a supportive and inclusive classroom environment.

The intervention comprised five sequential modules designed to support children's cognitive, emotional, and behavioral readiness for mathematics learning:

- Module 1: Body Awareness and Balance—Simple postures and breath-awareness exercises were used to help students recognize their bodies and improve balance.
- Module 2: Focus and Attention—Quiet-waiting practices, object-focused tasks, and rhythmic breathing activities were incorporated to enhance readiness for learning.
- Module 3: Calming and Emotion Regulation—Relaxation techniques, slowed breathing exercises, and guided imagery were used to alleviate mathematics-related anxiety.
- Module 4: Movement-Based Mathematical Preparation—Number sequences, directional tracking, and mental-clearing activities were performed alongside simple movement patterns to prepare students for problem-solving tasks.
- Module 5: Resilience, Self-Confidence, and Positive Academic Attitude—Strength-building yoga poses, positive self-talk practices, and self-efficacy-oriented learning activities were implemented.

Overall, the program was designed using a holistic framework to help students cope more effectively with cognitive and emotional challenges encountered during mathematics learning, strengthen focus and emotional regulation skills, manage mathematics anxiety, and become mentally prepared for problem solving. Additionally, it provided a safe, systematic, and child-friendly movement-based learning experience that increased students' physical activity levels.

### Data analysis

The statistical analyses were conducted using IBM SPSS Statistics Version 30.0. Prior to hypothesis testing, the dataset was screened for missing values, outliers, and compliance with the assumptions of parametric analyses. Normality was evaluated using the Kolmogorov–Smirnov and Shapiro–Wilk tests, together with the examination of skewness and kurtosis coefficients and visual inspection of histograms and Q–Q plots. The distributions of all continuous variables were considered sufficiently normal to justify the use of parametric statistical procedures.

Baseline equivalence between the experimental and control groups was examined using independent-samples *t*-tests. Changes from pre-test to post-test within each group were evaluated using paired-samples *t*-tests, whereas post-test differences between groups were assessed using independent-samples *t*-tests.

To examine intervention effects while controlling for baseline performance, separate one-way analyses of covariance (ANCOVA) were conducted for each outcome variable using the corresponding pre-test score as the covariate. Prior to conducting ANCOVA, the assumptions of linearity, homogeneity of regression slopes, homogeneity of variances, normality of residuals, and independence of observations were evaluated and were considered acceptable.

Because five primary outcome analyses were conducted (problem-solving, computation, total mathematics achievement, mathematics anxiety, and physical activity), a Bonferroni adjustment was applied to control the family-wise Type I error rate. Accordingly, statistical significance for the primary outcome analyses was interpreted using an adjusted alpha level of *p* < 0.01 (0.05/5). All statistically significant findings remained significant after this adjustment. Although the total mathematics score represents the arithmetic sum of the problem-solving and computation subscales and is therefore not statistically independent, it was included to facilitate comparison with previous studies reporting overall mathematics achievement. The primary interpretation of intervention effects focused on the individual subscale analyses, whereas the total score was considered a complementary summary outcome.

Effect sizes were reported to facilitate the interpretation of the practical significance of the findings. For independent-group comparisons, Cohen's d was calculated using pooled standard deviations, whereas within-group effect sizes were calculated using standardized mean differences for paired observations. ANCOVA results are reported using partial eta squared (partial η^2^). Effect sizes were interpreted according to conventional recommendations (61), where values of approximately 0.20, 0.50, and 0.80 represent small, medium, and large effects for Cohen's *d*, and 0.01, 0.06, and 0.14 represent small, medium, and large effects for partial η^2^.

An a priori power analysis conducted using G^*^Power indicated that the planned sample size was adequate to detect a large intervention effect with a statistical power of at least 0.80 at an alpha level of 0.05. Statistical significance was set at *p* < 0.05 for all analyses.

Although statistical analyses were performed at the individual student level, the intervention was implemented in two intact classrooms. Therefore, the findings should be interpreted with appropriate caution because classroom- and teacher-related factors could not be fully separated from the intervention effect.

Although statistical analyses were conducted at the individual student level, the intervention was implemented at the classroom level. Accordingly, the findings should be interpreted with appropriate caution because the hierarchical structure of the data could not be modeled with only one classroom per study condition.

### Ethical approval

This study was reviewed and approved by the Scientific Research and Publication Ethics Committee of Muş Alparslan University at its meeting held on 11 May 2022 (Meeting No. 7, Decision No. 59). The study was conducted in accordance with all principles set forth by the ethics committee, as well as the ethical standards outlined in the 1964 Declaration of Helsinki and its subsequent amendments. Informed consent was obtained from all participating students and their parents, participation was entirely voluntary, and participant confidentiality was rigorously maintained.

## Results

Prior to the intervention, independent-samples *t*-tests were performed to determine whether the experimental and control groups were comparable with respect to verbal problem-solving performance. Descriptive statistics and group comparisons are presented in Table 1.

The results indicated that no statistically significant differences existed between the experimental and control groups on any of the baseline mathematics measures. For the problem-solving subscale, the experimental group (M = 10.72, SD = 1.49) and the control group (M = 11.00, SD = 1.13) demonstrated comparable performance, *t*_(_46) = −0.73, *p* =0.469, *d* = −0.21. Likewise, no statistically significant difference was observed for computation scores, *t*_(_46) = 0.57, *p* = 0.570, with mean scores of 7.92 (SD = 0.81) and 7.78 (SD = 0.85) for the experimental and control groups, respectively. Total mathematics scores also did not differ significantly between the groups, *t*_(_46) = −0.32, *p* = 0.751, *d* = −0.09.

Overall, these findings indicate that the experimental and control groups were statistically comparable before the intervention across all baseline mathematics measures.

Changes in mathematics performance from pre-test to post-test were examined separately within each group using paired-samples *t*-tests. Independent-samples *t*-tests were subsequently conducted to compare post-test performance between the experimental and control groups. The descriptive statistics and statistical results are presented in Table 2.

**Table 2:** Baseline (pre-test) comparison of verbal problem-solving test scores.

Variable	Group	*n*	Mean	SD	*t*_(_46)	*p*	Cohen's d
Problem	Experimental	25	10.72	1.49	−0.73	0.469	−0.21
	Control	23	11.00	1.13			
Computation	Experimental	25	7.92	0.81	0.57	0.570	0.17
	Control	23	7.78	0.85			
Total	Experimental	25	18.64	1.73	−0.32	0.751	−0.09
	Control	23	18.78	1.31			

*n*, sample size; SD, standard deviation; Cohen's *d*, standardized mean difference.

Within the experimental group, statistically significant improvements were observed across all mathematics outcomes. Problem-solving scores increased from a mean of 10.72 at pre-test to 17.96 at post-test, *t* = 13.42, *p* < 0.001, *d* = 2.68. Computation scores increased from 7.92 to 12.60, *t* = 14.62, *p* < 0.001, *d* = 2.92, while total mathematics scores increased from 18.64 to 30.56, *t* = 15.98, *p* < 0.001, *d* = 3.20.

The control group also demonstrated statistically significant increases over time. Problem-solving scores increased from 11.00 to 11.91, *t* = 3.10, *p* = 0.005, computation scores increased from 7.78 to 9.09, *t* = 4.59, *p* < 0.001, and total mathematics scores increased from 18.78 to 21.00, *t* = 5.32, *p* < 0.001. The standardized effect sizes observed within the control group were smaller than those obtained for the experimental group.

Post-test comparisons further demonstrated statistically significant differences between the groups. Students in the experimental group obtained higher post-test scores than those in the control group for problem-solving (*t* = 12.61, *p* < 0.001, *d* = 3.64), computation (*t* = 11.24, *p* < 0.001, *d* = 3.25), and total mathematics performance (*t* = 14.60, *p* < 0.001, *d* = 4.22).

The descriptive statistics and inferential analyses are summarized in Table 3.

**Table 3:** Pre-test–post-test changes and between-group comparisons for the verbal problem-solving test.

Variable	Group	Pre-test mean	Post-test mean	*t* (within group)	*p*	Cohen's d	Post-test SD	*t* (between groups)	*p*	Cohen's d
Problem	Experimental	10.72	17.96	13.42	< 0.001	2.68	2.11			
	Control	11.00	11.91	3.10	0.005	0.65	0.95	12.61	< 0.001	3.64
Computation	Experimental	7.92	12.60	14.62	< 0.001	2.92	1.26			
	Control	7.78	9.09	4.59	< 0.001	0.96	0.85	11.24	< 0.001	3.25
Total	Experimental	18.64	30.56	15.98	< 0.001	3.20	2.84			
	Control	18.78	21.00	5.32	< 0.001	1.11	1.38	14.60	< 0.001	4.22

Within-group comparisons were conducted using paired-samples *t*-tests. Between-group comparisons were based on independent-samples *t*-tests using post-test scores. Cohen's *d* values represent standardized effect sizes.

To evaluate post-test differences while controlling for baseline performance, separate one-way analyses of covariance (ANCOVA) were conducted for problem-solving, computation, and total mathematics scores using the corresponding pre-test score as the covariate. Adjusted post-test means together with the ANCOVA statistics are presented in Table 3.

After adjustment for baseline performance, statistically significant group differences were observed for all mathematics outcomes. For the problem-solving subscale, the adjusted post-test mean was 17.95 for the experimental group and 11.92 for the control group. The group effect was statistically significant, *F*_(1, 45)_ = 153.18, *p* < 0.001, partial η^2^ = 0.773.

Similarly, the adjusted computation scores were 12.62 for the experimental group and 9.07 for the control group, yielding a statistically significant group effect, *F*_(1, 45)_ = 130.61, *p* < 0.001, partial η^2^ = 0.744.

For total mathematics performance, the adjusted post-test mean was 30.54 for the experimental group compared with 21.03 for the control group. The ANCOVA indicated a statistically significant group effect, *F*_(1, 45)_ = 218.15, *p* < 0.001, partial η^2^ = 0.829.

The adjusted means and ANCOVA statistics are presented in Table 4.

**Table 4:** ANCOVA results for verbal problem-solving performance.

Outcome	Adjusted mean (experimental)	Adjusted mean (control)	SS effect	SS error	df	MS effect	MS error	*F*	*p*	Partial η^2^
Problem	17.95	11.92	430.40	126.44	1.45	430.40	2.81	153.18	< 0.001	0.773
Computation	12.62	9.07	149.84	51.62	1.45	149.84	1.15	130.61	< 0.001	0.744
Total	30.54	21.03	1081.14	223.02	1.45	1081.14	4.96	218.15	< 0.001	0.829

ANCOVA was conducted using post-test scores as the dependent variable and the corresponding pre-test scores as the covariate. SS, sum of squares; MS, mean square; Partial η^2^, partial eta squared.

To evaluate changes in mathematics anxiety following the intervention, paired-samples *t*-tests were conducted within each group. Independent-samples *t*-tests were subsequently used to compare post-test scores between the experimental and control groups. The results are summarized in Table 5.

**Table 5:** Baseline (pre-test) comparison of mathematics anxiety scores.

Variable	Group	n	Mean	SD	t(46)	*p*	Cohen's d
Mathematics anxiety	Experimental	25	27.83	3.10	0.39	0.700	0.11
	Control	23	27.50	2.84			

*n*, sample size; SD, standard deviation; Cohen's *d*, standardized mean difference.

Within the experimental group, mathematics anxiety decreased significantly from a pre-test mean of 27.83 to a post-test mean of 22.42, *t* = −7.45, *p* < 0.001, *d* = −1.52. In contrast, although the control group showed a slight reduction in mathematics anxiety (27.50 to 25.96), this change was not statistically significant, *t* = −1.79, *p* = 0.087, *d* = −0.37.

Post-test comparisons revealed significantly lower mathematics anxiety scores in the experimental group than in the control group, *t* = −4.88, *p* < 0.001, corresponding to a large standardized mean difference (*d* = −1.41).

The descriptive statistics and inferential analyses are presented in Table 6.

**Table 6:** Pre-test–post-test changes and between-group comparisons for mathematics anxiety.

Group	Pre-test mean	Post-test mean	*t* (within group)	*p*	Cohen's d	Post-test SD	*t* (between groups)	*p*	Cohen's d
Experimental	27.83	22.42	−7.45	< 0.001	−1.52	2.34			
Control	27.50	25.96	−1.79	0.087	−0.37	2.68	−4.88	< 0.001	−1.41

Within-group comparisons were conducted using paired-samples *t*-tests. Between-group comparisons were based on independent-samples *t*-tests using post-test scores. Lower scores indicate lower mathematics anxiety.

To examine post-test mathematics anxiety while controlling for baseline differences, a one-way analysis of covariance (ANCOVA) was conducted using pre-test mathematics anxiety scores as the covariate. The adjusted post-test means and ANCOVA statistics are presented in Table 7.

**Table 7:** ANCOVA results for mathematics anxiety.

Outcome	Adjusted mean (experimental)	Adjusted mean (control)	SS effect	SS error	df	MS effect	MS error	*F*	*p*	Partial η^2^
Mathematics anxiety	22.42	25.96	149.90	290.78	1.45	149.90	6.46	23.20	< 0.001	0.340

ANCOVA was performed using post-test mathematics anxiety scores as the dependent variable and pre-test mathematics anxiety scores as the covariate. SS, sum of squares; MS, mean square; Partial η^2^, partial eta squared.

After controlling for baseline mathematics anxiety, a statistically significant group effect was observed. The adjusted mean mathematics anxiety score was 22.42 for the experimental group and 25.96 for the control group. The ANCOVA indicated that the difference between groups remained statistically significant, *F*_(1, 45)_ = 23.20, *p* < 0.001, with a partial eta squared of 0.340.

To determine whether the experimental and control groups were comparable with respect to physical activity before the intervention, an independent-samples *t*-test was conducted using the pre-test PAQ-C scores. The descriptive statistics and group comparisons are presented in Table 8.

**Table 8:** Baseline (pre-test) comparison of physical activity (PAQ-C) scores.

Variable	Group	*n*	Mean	SD	*t*_(_46)	*p*	Cohen's *d*
PAQ-C (pre-test)	Experimental	25	2.96	0.24	−0.44	0.665	−0.13
	Control	23	2.99	0.26			

PAQ-C, Physical Activity Questionnaire for Older Children; *n*, sample size; SD, standard deviation; Cohen's *d*, standardized mean difference.

As shown in Table 8, no statistically significant difference was observed between the experimental group (M = 2.96, SD = 0.24) and the control group (M = 2.99, SD = 0.26) prior to the intervention, *t*_(_46) = −0.44, *p* = 0.665, *d* = −0.13. The negligible effect size indicates that both groups reported comparable levels of physical activity at baseline.

To evaluate changes in physical activity following the intervention, paired-samples *t*-tests were conducted separately within each group. Independent-samples *t*-tests were subsequently performed to compare post-test physical activity levels between the experimental and control groups. The results are presented in Table 8.

Within the experimental group, PAQ-C scores increased significantly from a pre-test mean of 2.96 to a post-test mean of 4.04, *t* = 12.15, *p* < 0.001, *d* = 2.43. In contrast, the control group demonstrated only a small increase in PAQ-C scores (2.99 to 3.06), which was not statistically significant, *t* = 0.84, *p* = 0.412, *d* = 0.17.

Comparison of post-test scores revealed that the experimental group reported significantly higher physical activity levels than the control group, *t* = 11.53, *p* < 0.001, corresponding to a large standardized mean difference (*d* = 3.33).

The descriptive statistics and inferential analyses are summarized in Table 9.

**Table 9:** Pre-test–post-test changes and between-group comparisons for physical activity (PAQ-C).

Group	Pre-test mean	Post-test mean	*t* (within group)	*p*	Cohen's *d*	Post-test SD	*t* (between groups)	*p*	Cohen's *d*
Experimental	2.96	4.04	12.15	< 0.001	2.43	0.34			
Control	2.99	3.06	0.84	0.412	0.17	0.23	11.53	< 0.001	3.33

Within-group comparisons were conducted using paired-samples *t*-tests. Between-group comparisons were based on independent-samples *t*-tests using post-test scores. Cohen's *d* values represent standardized effect sizes.

To examine post-test physical activity levels while controlling for baseline physical activity, a one-way analysis of covariance (ANCOVA) was conducted using the pre-test PAQ-C score as the covariate. The adjusted post-test means and ANCOVA statistics are presented in Table 10.

**Table 10:** ANCOVA results for physical activity (PAQ-C).

Outcome	Adjusted mean (experimental)	Adjusted mean (control)	SS effect	SS error	df	MS effect	MS error	*F*	*p*	Partial η^2^
PAQ-C	4.03	3.06	11.32	3.89	1.45	11.32	0.086	130.90	< 0.001	0.744

ANCOVA was conducted using post-test PAQ-C scores as the dependent variable and pre-test PAQ-C scores as the covariate. SS, sum of squares; MS, mean square; Partial η^2^, partial eta squared.

After adjustment for baseline physical activity, the experimental group had a higher adjusted post-test mean PAQ-C score (M = 4.03) than the control group (M = 3.06). The ANCOVA indicated a statistically significant group effect, *F*_(1, 45)_ = 130.90, *p* < 0.001, with a partial eta squared of 0.744.

## Discussion

The present study examined whether integrating a structured yoga-based mind–body movement program into regular mathematics lessons was associated with changes in third-grade students' mathematical performance, mathematics anxiety, and self-reported physical activity. Compared with students who received standard mathematics instruction, those who participated in the yoga-integrated program demonstrated higher post-test mathematics scores, lower mathematics anxiety, and higher PAQ-C scores. After controlling for baseline performance using ANCOVA, these differences remained statistically significant. Collectively, these findings suggest that embedding structured movement activities within academic instruction may represent a promising educational approach for supporting cognitive, emotional, and behavioral outcomes in elementary school settings. However, the findings should be interpreted with appropriate caution. The intervention was implemented in one intact classroom and compared with a single control classroom, meaning that treatment assignment was completely confounded with classroom membership, teacher characteristics, instructional context, peer interactions, and daily classroom routines. Although baseline differences were statistically controlled using ANCOVA, the hierarchical structure of the data could not be explicitly modeled because only one classroom represented each study condition. Accordingly, the observed differences should be interpreted as evidence of an association under the specific conditions of the present study rather than as definitive evidence of a causal intervention effect.

The observed improvements in mathematics performance are consistent with an expanding body of literature indicating that movement-based educational approaches and school-based mind–body interventions may support academic learning by facilitating attentional regulation, executive functioning, and classroom engagement (46–49). Executive functions including inhibitory control, working memory, and cognitive flexibility play fundamental roles in mathematical reasoning, problem solving, and computational accuracy. Although these cognitive processes were not directly assessed in the present study, previous research suggests that yoga-based activities may create classroom conditions that facilitate the use of these executive resources during learning. Consequently, the improvements observed in mathematics achievement may reflect one of several plausible pathways through which structured movement activities support academic performance rather than evidence for a single underlying mechanism.

Similarly, the reduction in mathematics anxiety observed among students in the intervention group aligns with previous evidence demonstrating that school-based yoga and other mind–body programs may contribute to improved emotional regulation and reduced anxiety symptoms in children (50, 51). Importantly, the present study did not directly measure physiological or neuropsychological mechanisms such as cortisol regulation, autonomic nervous system activity, interoceptive awareness, or emotion regulation. Therefore, these mechanisms should be regarded as theoretical explanations derived from previous literature rather than conclusions supported directly by the present findings. Future studies incorporating physiological markers and validated measures of emotional regulation would provide a more direct examination of these potential pathways.

The increase in self-reported physical activity is also broadly consistent with previous investigations indicating that movement-integrated school programs can increase children's participation in physical activity during the school day (52, 53). However, this outcome should be interpreted cautiously because physical activity was assessed using the Physical Activity Questionnaire for Older Children (PAQ-C), a self-report instrument that may be influenced by recall bias, social desirability, and participants' awareness of group allocation. Because children in the intervention group were aware that they were participating in a novel movement-based program, some degree of response bias cannot be excluded. Future research should therefore complement self-report measures with objective indicators of physical activity, such as accelerometers or wearable activity monitors, to strengthen the validity of conclusions regarding changes in moderate-to-vigorous physical activity.

From a theoretical perspective, the findings are compatible with embodied cognition frameworks, which propose that learning is supported through interactions among perception, action, and cognitive processing (54–56). Rather than viewing mathematical learning as exclusively abstract and symbolic, embodied perspectives suggest that sensorimotor experiences may facilitate conceptual understanding and sustained engagement during learning activities. Integrating yoga-based movement into mathematics instruction may therefore represent one practical approach for embedding embodied learning principles within everyday classroom practice. Nevertheless, because no measures of embodied cognition, executive functioning, or attentional processes were collected, the present findings cannot determine which theoretical mechanisms primarily account for the observed improvements. These explanations should therefore be considered interpretive rather than confirmatory.

From a curriculum and instructional design perspective, the present findings may also contribute to the growing body of research supporting integrated and holistic approaches to education. Contemporary curriculum frameworks increasingly emphasize that cognitive, emotional, social, and physical development should not be addressed as isolated educational targets but rather as interconnected dimensions of children's learning experiences. Whole-child education models similarly advocate the integration of academic learning with students' physical health, emotional wellbeing, and social development within routine educational practices. In this context, movement-integrated learning may be conceptualized as a form of curriculum integration that enables schools to pursue health promotion and academic objectives simultaneously rather than treating them as competing priorities within limited instructional time. The yoga-integrated mathematics activities implemented in the present study may therefore be viewed not only as a classroom intervention but also as an example of holistic curriculum design aimed at supporting whole-child development through interdisciplinary learning experiences. From a curriculum development perspective, these findings suggest that movement-based and self-regulation-oriented learning experiences could be systematically embedded within mathematics curricula and other subject areas as part of broader school-wide educational programs designed to promote both academic achievement and student wellbeing (15, 21, 57).

One noteworthy aspect of the present study is that the intervention was implemented directly within routine mathematics lessons rather than as a separate physical education or extracurricular activity. This ecological approach increases the practical relevance of the findings because it reflects conditions that schools may realistically implement without substantial curriculum restructuring. At the same time, caution is warranted when attributing the observed benefits specifically to yoga. The intervention incorporated multiple components, including structured movement, novelty, regular activity breaks, and sustained teacher guidance. Consequently, it is not possible to determine the relative contribution of yoga-specific elements compared with more general influences such as increased movement opportunities, enhanced classroom engagement, or additional teacher attention.

The experimental group participated in approximately 60 min of additional structured movement activities per week that were integrated into routine mathematics lessons, whereas the control group continued to receive standard mathematics instruction without an equivalent movement-based or attention-matched activity. Consequently, the observed differences may reflect not only yoga-specific components but also the educational benefits of additional movement opportunities, scheduled activity breaks, increased teacher attention, or the novelty associated with participation in a structured intervention. Therefore, the present findings should not be interpreted as demonstrating effects that are unique to yoga. Future randomized controlled trials should include active comparison conditions involving movement activities of comparable duration and teacher interaction but without yoga-specific elements to isolate the specific contribution of yoga-based practices.

An additional methodological consideration concerns the instructional context in which the intervention was implemented. The yoga-integrated program was delivered by the experimental classroom teacher, whereas the control classroom continued mathematics instruction with its regular classroom teacher. Consequently, teacher-related characteristics, instructional style, classroom climate, and peer interactions were inherently confounded with treatment assignment. Therefore, the observed differences cannot be attributed exclusively to the yoga-integrated program, as it is not possible to disentangle intervention effects from potential classroom- or teacher-level influences. This issue represents a more important threat to internal validity than the absence of individual randomization itself. Future studies should therefore include multiple classrooms across several schools and employ cluster-randomized or multilevel designs to distinguish intervention effects from teacher- and classroom-related influences.

Several methodological considerations should also be acknowledged when interpreting the findings. First, the study employed a quasi-experimental design using two intact classrooms, with one classroom assigned to the intervention and the other serving as the comparison group. Consequently, classroom characteristics, teacher-related factors, peer dynamics, and other contextual influences were completely confounded with treatment assignment. Although baseline equivalence was established across the principal outcome variables and statistical adjustment was performed using ANCOVA, residual confounding cannot be completely excluded. Accordingly, causal interpretations should remain cautious. Future investigations should incorporate multiple classrooms across several schools and use cluster randomization together with multilevel statistical analyses to better account for the hierarchical structure of educational data.

Second, although statistically significant improvements were observed across several outcomes, the magnitude of the observed effects should be interpreted within the context of the study design. Educational interventions implemented under relatively controlled conditions may yield larger effects than those observed in broader school populations, particularly when sample sizes are modest and implementation fidelity is high. Replication across larger and more diverse samples is therefore essential before generalizing these findings to wider educational settings.

Third, although baseline equivalence was established statistically, the relatively modest sample size drawn from a single public primary school may limit the generalizability of the findings to other educational contexts, grade levels, and cultural settings. Replication across geographically diverse schools and more heterogeneous student populations would provide stronger evidence regarding the external validity of yoga-integrated mathematics instruction.

Finally, the present study evaluated outcomes immediately following the 10-week intervention. Consequently, the durability of the observed improvements remains unknown. Longitudinal follow-up studies are needed to determine whether gains in mathematics achievement, reductions in mathematics anxiety, and changes in physical activity are maintained over time and whether continued implementation is necessary to sustain these benefits.

Despite these limitations, the study contributes to the emerging literature on movement-integrated learning by examining an educational approach that has received relatively limited empirical attention. Rather than delivering yoga as an isolated wellness activity, the intervention embedded structured mind–body practices directly within routine mathematics instruction, thereby extending previous research that has generally examined physical activity and academic learning as separate educational domains (58–60). Although additional evidence from larger randomized and multi-school studies is needed, the present findings suggest that integrating structured movement experiences into mathematics lessons may represent a feasible and educationally meaningful strategy for supporting children's academic engagement, emotional wellbeing, and classroom participation. Future research incorporating active control groups, objective measures of physical activity, executive-function assessments, physiological indicators, and multilevel experimental designs will be important for clarifying the mechanisms underlying these associations and establishing the effectiveness of yoga-integrated mathematics instruction across diverse educational settings.

## Conclusion

The present study examined whether integrating a structured yoga-based mind–body movement program into regular mathematics lessons was associated with improvements in mathematical performance, mathematics anxiety, and self-reported physical activity among third-grade students. Compared with standard mathematics instruction, participation in the integrated program was associated with higher mathematics achievement, lower mathematics anxiety, and higher reported physical activity at post-test. These findings suggest that incorporating structured movement experiences within routine academic instruction may represent a feasible educational strategy for simultaneously supporting cognitive, emotional, and behavioral aspects of learning. Importantly, the findings should be interpreted cautiously because the quasi-experimental design does not permit strong causal inferences. In particular, the intervention was implemented in one intact classroom and compared with a single control classroom, meaning that classroom context, teacher characteristics, instructional practices, and peer interactions were inherently confounded with treatment assignment. Accordingly, the findings should be interpreted within the context of a classroom-based quasi-experimental design rather than as definitive evidence of causal intervention effects. Nevertheless, the study contributes to the emerging literature by examining yoga-based mind–body practices as an integrated component of mathematics instruction rather than as a stand-alone physical activity program. This perspective extends current research on movement-integrated learning and highlights the potential educational value of embedding structured self-regulation activities within everyday classroom practice. Future research should replicate these findings using larger multi-school samples, active comparison groups, objective measures of physical activity (e.g., accelerometry), and longitudinal follow-up assessments. In addition, future studies should employ cluster-randomized controlled trials involving multiple classrooms across several schools to permit multilevel statistical modeling of student- and classroom-level variance. Such designs would allow researchers to distinguish intervention effects from classroom-, teacher-, and school-level influences, thereby providing stronger evidence regarding the effectiveness of yoga-integrated mathematics instruction and clarifying the specific contribution of yoga-based practices beyond the general benefits of movement-integrated learning.

## Strengths and limitations

Several strengths should be considered when interpreting the findings. First, the intervention was embedded directly within routine mathematics lessons rather than delivered as a separate extracurricular activity, increasing the ecological validity and practical relevance of the study. Second, multiple educational outcomes—including mathematics achievement, mathematics anxiety, and physical activity—were evaluated, providing a broader perspective on the potential educational implications of movement-integrated instruction. Third, intervention implementation was monitored throughout the study using standardized procedures, supporting consistency in program delivery.

At the same time, several limitations should be acknowledged. First, the study employed a quasi-experimental design involving two intact classrooms, with one classroom assigned to the intervention and one to the comparison condition. Consequently, classroom characteristics, teacher-related influences, peer interactions, and other contextual factors were completely confounded with treatment assignment. Although baseline equivalence was established for the principal outcome measures and statistical adjustment was performed using ANCOVA, these procedures cannot fully eliminate potential clustering effects or residual confounding. Therefore, causal interpretations should be made cautiously.

Second, the comparison group received standard mathematics instruction and did not participate in an attention-matched movement activity. As a result, some of the observed differences may reflect non-specific factors such as increased movement opportunities, classroom novelty, or additional teacher attention rather than yoga-specific components of the intervention. Future studies should include active control conditions to isolate the unique contribution of yoga-based practices.

Third, physical activity was assessed using the Physical Activity Questionnaire for Older Children (PAQ-C), which relies on self-report. Because participants were aware of their group allocation, response bias and social desirability cannot be excluded. Future investigations should therefore incorporate objective indicators of physical activity, such as accelerometers or wearable activity monitors.

Fourth, although statistically significant improvements were observed across all outcomes, the relatively modest sample size drawn from a single school limits the generalizability of the findings. Replication across larger and more diverse educational settings is necessary to determine whether similar results can be achieved under different instructional conditions.

Finally, outcomes were assessed immediately after completion of the 10-week intervention. Consequently, the long-term sustainability of the observed improvements remains unknown. Longitudinal follow-up studies are required to determine whether gains in mathematics achievement, reductions in mathematics anxiety, and increases in physical activity persist over time and whether continued implementation is necessary to maintain these benefits.

## Data Availability

The raw data supporting the conclusions of this article will be made available by the authors, without undue reservation.
